# *Arabidopsis* CCoAOMT1 Plays a Role in Drought Stress Response via ROS- and ABA-Dependent Manners

**DOI:** 10.3390/plants10050831

**Published:** 2021-04-21

**Authors:** Hyun Jin Chun, Lack Hyeon Lim, Mi Sun Cheong, Dongwon Baek, Mi Suk Park, Hyun Min Cho, Su Hyeon Lee, Byung Jun Jin, Dong Hyeon No, Ye Jin Cha, Yong Bok Lee, Jong Chan Hong, Dae-Jin Yun, Min Chul Kim

**Affiliations:** 1 Institute of Agriculture and Life Science, Gyeongsang National University, Jinju 52828, Korea; hj_chun@hanmail.net (H.J.C.); mscheong@gnu.ac.kr (M.S.C.); yblee@gnu.ac.kr (Y.B.L.); 2 Division of Applied Life Science (BK21 Four), Gyeongsang National University, Jinju 52828, Korea; dlafkrgus@gnu.ac.kr (L.H.L.); hmcho86@gnu.ac.kr (H.M.C.); leesuhyeon86@gmail.com (S.H.L.); scv5789@naver.com (B.J.J.); no0513w@naver.com (D.H.N.); cdw3280@naver.com (Y.J.C.); 3 Plant Molecular Biology and Biotechnology Research Center, Gyeongsang National University, Jinju 52828, Korea; dw100@hanmail.net (D.B.); misugip@hanmail.net (M.S.P.); jchong@gnu.ac.kr (J.C.H.); 4 Department of Biomedical Science and Engineering, Konkuk University, Seoul 05029, Korea; djyun@konkuk.ac.kr

**Keywords:** *CCoAOMT1*, drought, abscisic acid (ABA), reactive oxygen species (ROS)

## Abstract

Plants possess adaptive reprogramed modules to prolonged environmental stresses, including adjustment of metabolism and gene expression for physiological and morphological adaptation. *CCoAOMT1* encodes a caffeoyl CoA O-methyltransferase and is known to play an important role in adaptation of *Arabidopsis* plants to prolonged saline stress. In this study, we showed that the *CCoAOMT1* gene plays a role in drought stress response. Transcript of *CCoAOMT1* was induced by salt, dehydration (drought), and methyl viologen (MV), and loss of function mutants of *CCoAOMT1*, *ccoaomt1-1,* and *ccoaomt1-2* exhibit hypersensitive phenotypes to drought and MV stresses. The *ccoaomt1* mutants accumulated higher level of H_2_O_2_ in the leaves and expressed lower levels of drought-responsive genes including *RD29B*, *RD20*, *RD29A*, and *ERD1,* as well as *ABA3 3* and *NCED3* encoding ABA biosynthesis enzymes during drought stress compared to wild-type plants. A seed germination assay of *ccoaomt1* mutants in the presence of ABA also revealed that *CCoAOMT1* functions in ABA response. Our data suggests that *CCoAOMT1* plays a positive role in response to drought stress response by regulating H_2_O_2_ accumulation and ABA signaling.

## 1. Introduction

Sessile plants are continuously exposed to external environmental stresses that threaten their survival. Plants adapt or survive in extreme environmental conditions [1] by evolving mechanisms that arrest growth and development under stresses and resume coming back in favorable conditions [2]. These mechanisms are involved in the modulating of the various processes in the morphological, anatomical, cellular, and molecular levels while responding to stimuli and integrating internal and external signals [3].

Since plant growth requires concerted water uptake and irreversible cell wall expansion to enlarge cells at the cellular level [4], water deficiency as a type of environmental stress, including drought, dehydration, osmotic, and salinity, can result in the reduction of plant growth. The plant cell wall is considered a protective barrier with a complex structure composed of cellulose microfibrils, non-cellulose polysaccharides, and lignin, all of which determine cell size and shape through the mechanical control of cell expansion [4]. An increase in the elasticity of the cell wall can contribute to the maintenance of cell turgor in response to environmental stresses [5]. This causes the structure of the plant cell wall and its contents to change following biotic and abiotic stresses [6]. For example, the various enzymatic reactions caused by drought stress involved in modified plant cell walls include stiffening, increasing in lignin content, and decreasing cell expansion to prevent water loss [7]. It is evident that lignin triggers cell wall rigidification and growth arrest in the later stage of drought stress, which could lead to a loss of productivity [5,8].

Drought stress is one of the major abiotic stresses during plant cultivation [9] and consequently enhances reactive oxygen species (ROS) production, which activates a signal to trigger acclamatory/defense response by a specific signal transduction pathway, such as H_2_O_2_ [10]. H_2_O_2_ plays a role as a secondary messenger in an ROS signal transduction pathway, since H_2_O_2_ (1) is a very stable ROS with the longest half-life, (2) easily diffuses inside of the cell, and (3) can be readily metabolized by an efficient cellular antioxidant system [11]. ROS have a dual effect under abiotic stress conditions that depend on their overall cellular amount [10,12]. If ROS are kept at relatively low levels, they are likely to function as components of a stress-signaling pathway, triggering stress defenses/acclimation responses. However, reaching a high level up to uncontrolled ROS cascades, which damages the cellular membrane and other cellular components, results in oxidative stress and eventually cell death [12].

In terms of plant stress hormone, the de novo biosynthesis of abscisic acid (ABA) is induced during abiotic stresses, and increased ABA level plays an important role in conferring tolerance against the water-deficit conditions such as drought and osmotic stresses [13,14]. Under drought stress, ABA upregulates the expression of many stress-responsive genes [15], minimizes the water loss through limited transpiration [16], and involves synthesizing of osmoprotectants and antioxidant enzymes [17]. Although ABA is a critical component for drought stress tolerance, the mechanism of drought stress responses follows two distinct pathways, either ABA-dependent or ABA-independent pathway [18]. As molecular responses, transcription factors determine the activation or repression of response pathways including either ABA-dependent or ABA-independent stress response [19]. These responsive genes are well-identified for molecular markers and have been used as a molecular tool to understand ABA responsiveness; for instance, the expression of *RD29B* and *RD20* is ABA/drought-responsive in an ABA-dependent pathway and that of *RD29A* and *ERD1* is drought-responsive in an ABA-independent pathway [18,19].

*CCoAOMT1* (At4g34050) encodes an enzyme synthesizing feruloyl CoA from caffeoyl CoA towards guaiacyl (G) and sinapyl (S) lignin biosynthesis [20]. The T-DNA inserted *Arabidopsis* mutants of *CCoAOMT1*, *ccoaomt1-1* (CS345826), and *ccoaomt1-2* (SALK_151507) exhibited a reduced amount of G monomer of lignin, but higher level of S and H (*p*-hydroxyohenyl) monomer, supporting that *CCoAOMT1* plays a key role in lignin biosynthesis [20,21]. In addition, these *ccoaomt1* mutants revealed hypersensitive phenotype upon salt stress by showing an inhibition of primary root elongation [22], suggesting that *CCoAOMT1* plays a role in salt stress adaptation by altering lignin biosynthesis with plant cell wall.

Recently, accumulating information showed that *CCoAOMT1* plays an important role for enhancing lignification and being tolerant to salt stress; however, a role of *CCoAOMT1* in response to other abiotic stresses including drought, ROS, and stress hormone ABA is still elusive. Here, we demonstrated that *CCoAOMT1* plays a role in responding to drought stress through both ROS and ABA signaling. Compared to wild-type (WT) plants, *ccoaomt1* plants exhibited drought stress-sensitive phenotype with higher water-loss, higher level of H_2_O_2_ accumulation, and lower expression of drought/ABA stress-response genes, such as *RD29B*, *RD20*, *RD29A*, *ERD1, NCED3,* and *ABA3,* during drought stress. In addition, we confirmed that *CCoAOMT1* plays a role in an ROS and ABA signaling by showing that *ccoaomt1* mutants were hypersensitive to MV stress and exhibited less delayed seed germination in the presence of ABA compared to WT.

## 2. Results

### 2.1. Arabidopsis CCoAOMT1 Is Involved in the Abiotic Stress Response

**Figure 1:**
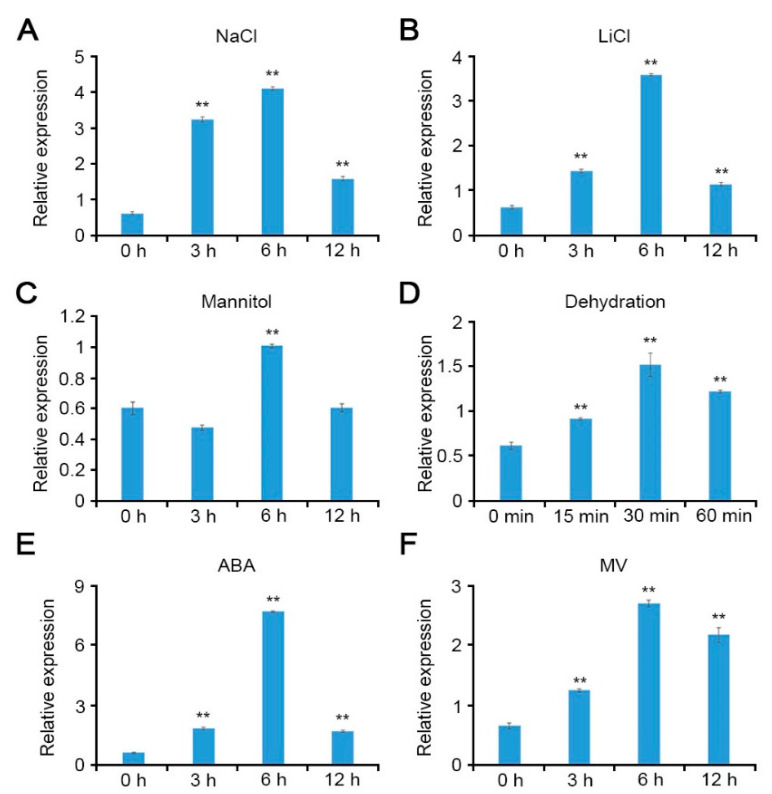
Transcriptional responses of *CCoAOMT1* toward various abiotic stresses. Relative transcript levels of *CCoAOMT1* were analyzed via quantitative real time-PCR (qRT-PCR). Total RNAs were extracted from 10-day-old *Arabidopsis* seedlings grown on 1/2 MS medium treated with (**A**) 100 mM NaCl, (**B**) 10 mM LiCl, (**C**) 100 mM mannitol, (**D**) dehydration, (**E**) 100 µM abscisic acid (ABA), or (**F**) 10 µM methyl viologen (MV) at the indicated time points. Expression of *TUBULIN2* was used for normalization. Bars represent mean ± SD of three biologic replicates with three technical replicates each. Asterisks represent significant differences from the control (0 h; ** *p* < 0.01, Student’s *t*-test).

To examine the potential role of CCoAOMT1 not only in salt stress, but other abiotic stresses, we analyzed expression of the *CCoAOMT1* gene under various abiotic stress conditions. Quantitative real time-PCR (qRT-PCR) was performed using 10-day-old *Arabidopsis* seedlings, which were treated with 100 mM NaCl, 10 mM LiCl, 100 mM mannitol, or dehydration, respectively, during the indicated time. The gene expression of *CCoAOMT1* was significantly induced by various abiotic stresses (Figure 1A–D). Furthermore, the *CCoAOMT1* gene was also responsive to 100 µM ABA and 10 µM MV treatment (Figure 1E,F). Methyl viologen (MV; *N*,-*N*′-dimethyl-4,-4′-bipyridinium dichloride) induces ROS production and is used for an experimental tool to understand ROS signaling and processing [23]. In detail, the *CCoAOMT1* expression increased and peaked at a 6 h time point (for NaCl, LiCl, mannitol, ABA, and MV) and tended to decrease during a later testing period. These results indicated that the *CCoAOMT1* gene is involved in ABA and ROS signaling and abiotic stress responses, such as salt and drought stresses.

### 2.2. Loss of CCoAOMT1 Function Exhibits a Sensitive Phenotype to Drought Stress by Accumulating a Higher Level of H_2_O_2_

**Figure 2:**
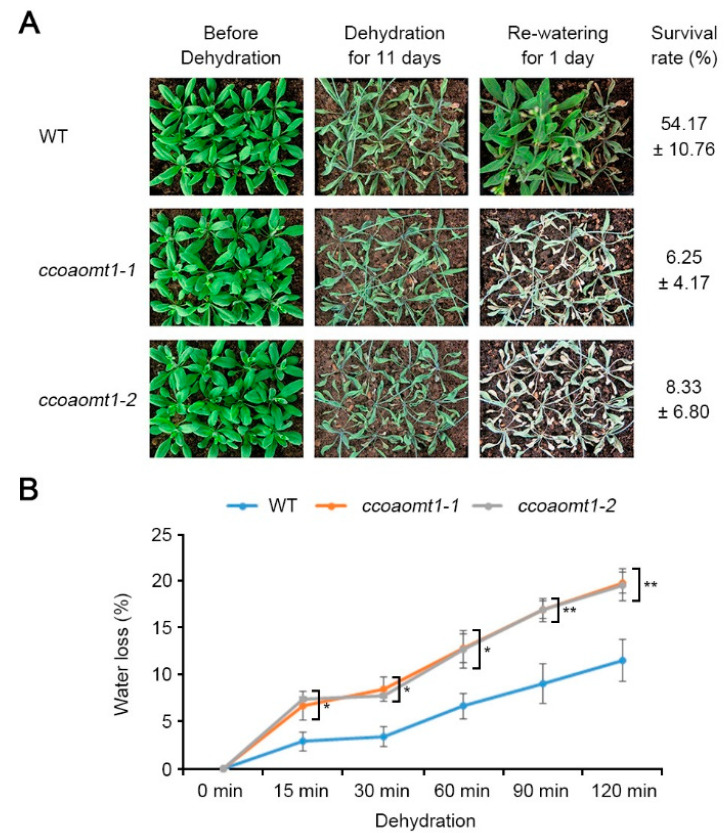
Hypersensitive phenotypes of *ccoaomt1* mutants to dehydration stress. (**A**) Morphological phenotype before and after dehydration stress treatment. Watering of soil-grown two-week-old wild-type (WT) and *ccoaomt1* mutants (*ccoaomt1-1* and *ccoaomt1-2*) was stopped for 11 days and survival rate was calculated at 1 day after rewatering and photos were taken. (**B**) Water loss of WT and *ccoaomt1* mutants during dehydration stress treatment. Water loss was calculated with percentage value (%) from the weight loss versus initial fresh weight from three-week-old plants. The quantitative values indicated means ± SD of three independent experiments. Asterisks represent significant differences from the WT (*, *p* < 0.05; **, *p* < 0.01; Student’s *t*-test).

Given that gene expression of *Arabidopsis CCoAOMT1* is upregulated during dehydration stress, we tested if *CCoAOMT1* affects drought stress response. To test this, we analyzed phenotypes of two-week-old WT (Col-0) plants and two *ccoaomt1* mutant lines including *ccoaomt1-1* and *ccoaomt1-2* during dehydration by stopping watering for 11 days and rewatering (Figure 2A). In contrast to WT plants, the *ccoaomt1-1* and *ccoaomt1-2* plants did not recover after rewatering. Then the survival rates (%) showed that both *ccoaomt1* mutants exhibited reduced values, 6.25 ± 4.17% for *ccoaomt1-1* and 8.33 ± 6.80% for *ccoaomt1-2*, compared with 54.17 ± 10.76% of WT (Figure 2A). This result indicated that loss of *CCoAOMT1* function shows a hypersensitive phenotype to drought stress, suggesting that *CCoAOMT1* is required during drought stress response.

To further verify drought-sensitive phenotype of *ccoaomt1* mutants, we examined the water loss during dehydration. To obtain water loss value (%), the aerial part of 3-week-old seedlings were detached and weighed at indicated time points, and the loss of fresh weight (%) was calculated (Figure 2B). As expected, both mutant plants exhibited significantly higher water loss value than WT. In addition, the slope of a water loss curve of mutant plants was higher than that of WT, indicating that *ccoaomt1* plants exhibit higher water loss. This information suggested that CCoAOMT1 is involved in controlling water loss under certain environmental conditions. Drought-sensitive phenotype of *ccoaomt1* plants was consistent with our previous study showing that *ccoaomt1-1* and *ccoaomt1-2* are sensitive to salt stress with suppressed primary root growth under saline condition (125 mM NaCl) [22]. Salt stress possesses two different aspects, ionic stress and osmotic stress, both of which subsequently cause dehydration and water deficit in the cellular level [24]. Taken together, we suggested that *CCoAOMT1* could be involved at least in dehydration stress-mediated water deficit response pathway.

**Figure 3:**
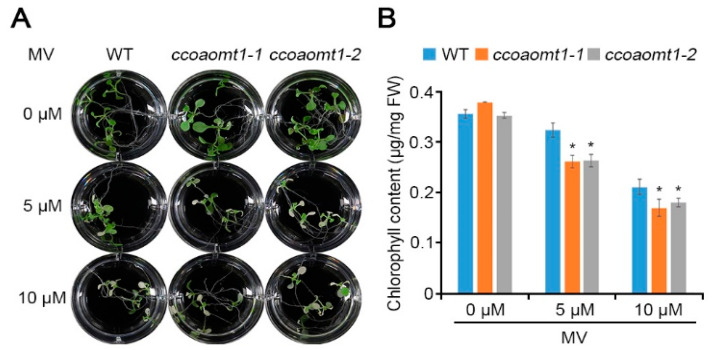
Hypersensitive phenotype of *ccoaomt1* mutants under MV stress conditions. (**A**) Morphological phenotype of WT and *ccoaomt1* mutants (*ccoaomt1-1* and *ccoaomt1-2*). Ten-day-old seedlings were grown on MS medium in the presence of indicated concentrations of MV for two days; 0 µM, 5 µM, and 10 µM MV. (**B**) Chlorophyll contents. The quantitative values indicated means ± SD with five seedlings from three independent experiments. Asterisks represent significant differences from the WT (*, *p* < 0.05; Student’s *t*-test).

To show that *CCoAOMT1* is upregulated during MV stress (Figure 1F), we further tested MV stress response of *ccoaomt1* mutants (Figure 3). First, 10-day-old seedlings were treated with 5 µM and 10 µM MV, which showed that *ccoaomt1* mutant plants were hypersensitive to MV with lower level of chlorophyll than WT. This result suggests that *CCoAOMT1* is involved in ROS-mediated plant responses to abiotic stresses.

**Figure 4:**
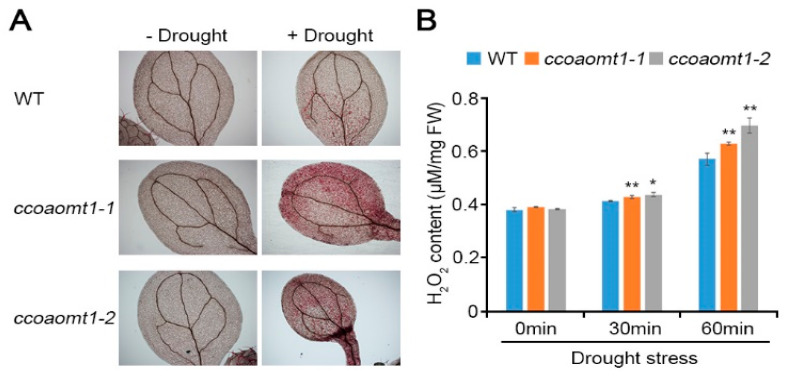
H_2_O_2_ accumulation in *ccoaomt1* mutants by drought stress. (**A**) DAB staining of drought-stressed leaves. The leaves of 10-day-old seedlings of WT and *ccoaomt1* mutants were stained with 3,3′-Diaminobenzidine (DAB) before and after drought stress (for 60 min). The red color represents insoluble ROS products, which hydrogen peroxide (H_2_O_2_) reacts with DAB. (**B**) Quantified H_2_O_2_ content. Internal H_2_O_2_ production assays were performed using WT and *ccoaomt1* mutants and the fluorescence intensity was measured with the range excitation at 530 nm and emission at 590 nm. The quantitative values indicated means ± SD of three independent experiments. Asterisks represent significant differences from the WT (*, *p* < 0.05; **, *p* < 0.01; Student’s *t*-test).

We found that *CCoAOMT1* is involved in plant responses to drought and MV stresses. To understand ROS regulation during drought stress, we measured H_2_O_2_ accumulation by drought stress (Figure 4). DAB staining, which forms an irreversible red-brown product with reacting H_2_O_2_, showed that *ccoaomt1* mutants accumulated higher level of H_2_O_2_ than WT during drought stress. This result indicates that *CCoAOMT1* is involved in regulation of H_2_O_2_ accumulation during drought stress, and further suggests that *CCoAOMT1* functions in drought stress through ROS signaling in drought stress response.

### 2.3. CCoAOMT1 Functions in Drought Response by Modulating Both ABA-Dependent and ABA-Independent Pathways

**Figure 5:**
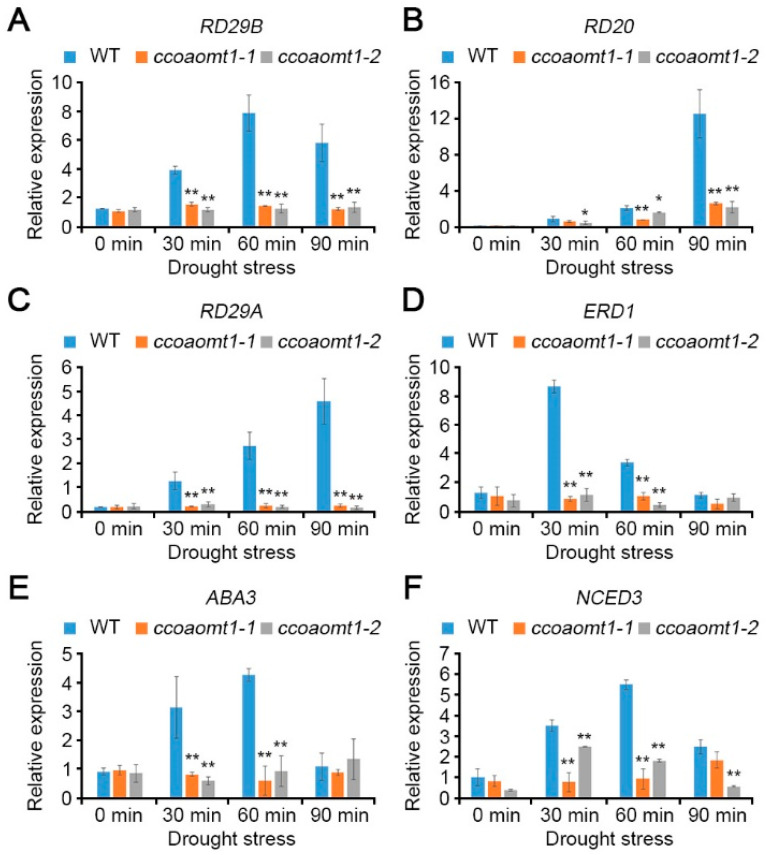
The mutation in *CCoAOMT1* gene affected the expression of ABA biosynthesis genes during drought stress. The relative transcript levels of *RD29B* (**A**), *RD20* (**B**), *RD29A* (**C**), *ERD1* (**D**), *ABA3* (**E**), and *NCED3* (**F**) were analyzed in WT and *ccoaomt1* mutants (*ccoaomt1-1* and *ccoaomt1-2*) using qRT-PCR. Total RNAs were extracted from 10-day-old seedlings treated with drought stress for 0, 30, 60, and 90 min, respectively. Expression of genes was normalized to that of *TUBULIN2*. Bars represent mean ± SD of three biological replicates with three technical replicates each. Asterisks represent significant differences from the WT (*, *p* < 0.05; **, *p* < 0.01; Student’s *t*-test).

Based on our observation that *CCoAOMT1* is responsive to ABA and drought stresses, we performed qRT-PCR to understand the role of *CCoAOMT1* in ABA signaling during drought stress responses. We initially exploited the expression patterns of drought stress-responsive genes involved in both ABA-dependent and ABA-independent pathways in *ccoaomt1* mutants. All tested responsive genes functioning not only in ABA-dependent pathway, such as *RD29B*, *RD20*, and ABA biosynthesis pathway (including *ABA3* and *NCED3)*, but also in ABA-independent pathway, such as *RD29A* and *ERD1*, were highly upregulated in WT plants under dehydration (Figure 5) [19]. Not surprisingly, both *ccoaomt1-1* and *ccoaomt1-2* plants showed downregulated expressions of six representative responsive genes upon dehydration compared to WT in a significant way (Figure 5). This result indicates that both *ccoaomt1-1* and *coaomt1-2* mutants were less tolerant than WT upon dehydration stress. The ABA-dependent pathway is involved in the de novo biosynthesis of ABA, which can increase ABA levels in abiotic stresses [14,25]. *NCED3* encodes 9-cis-epoxycarotenoid dioxygenase, which plays a rate-limiting role, and *ABA3* encodes molybdenum cofactor sulfurase, which catalyzes the last step for ABA biosynthesis. Both genes are highly induced by osmotic stress and contribute to increasing ABA level and tolerance to drought stress [26,27]. The reduced expression of *NCED3* and *ABA3* in *ccoaomt1-1* and *ccoaomt1-2* plants (Figure 5E,F) further revealed that *ccoaomt1* mutants were not able to induce ABA biosynthesis upon dehydration compared with WT, resulting in a lower level of ABA than WT.

### 2.4. CCoAOMT1 Gene Is Responsive to ABA Stress

**Figure 6:**
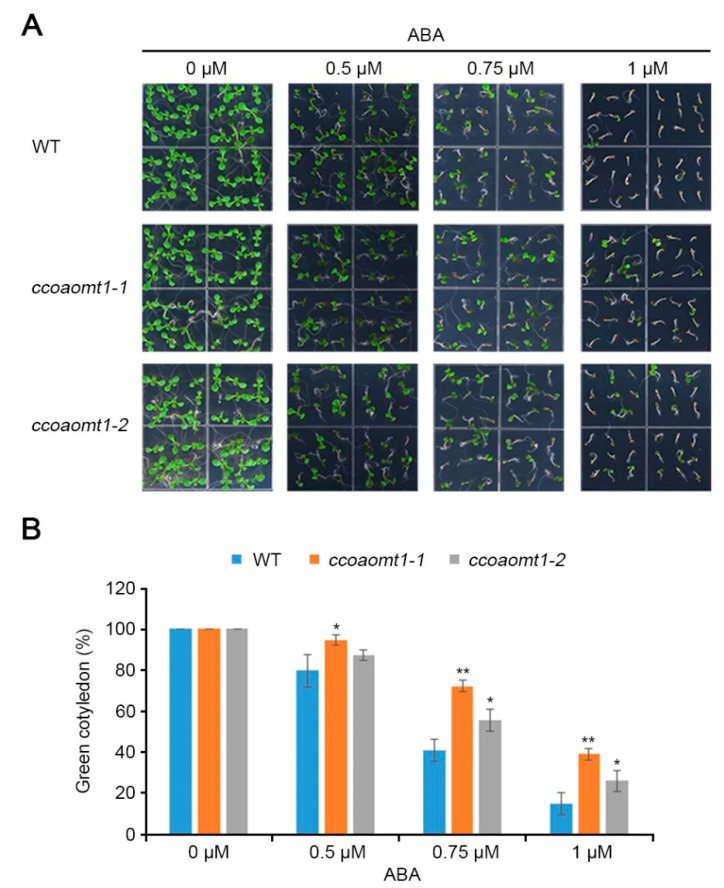
ABA-responsive phenotype of *ccoaomt1* mutants during seed germination in the presence of ABA. (**A**) Morphological phenotype of WT and *ccoaomt1* mutants (*ccoaomt1-1* and *ccoaomt1-2*). Seeds were germinated on an MS medium without (0 µM ABA) or with 0.5 µM, 0.75 µM, and 1 µM ABA, respectively. (**B**) Quantitative value of green cotyledons (%) of WT and *ccoaomt1* mutants. Opened green cotyledons were counted from indicated concentrations of ABA (0, 0.5, 0.75, and 1.0 μM) at 5 days after treatment. The values indicated means ± SD of 36 seeds from three independent experiments. Asterisks represent significant differences from the WT (*, *p* < 0.05; **, *p* < 0.01; Student’s *t*-test).

ABA acts in plant growth and development such as seed germination and dormancy in addition to adaptation to environmental stresses [25]. Seed germination is the physiological process that begins with water uptake by the seed and ends in showing an opening of green cotyledons by following decreased ABA levels [28,29]. Given that *CCoAOMT1* acts in the ABA signaling during drought stress response by regulating the expression of ABA responsive genes, including *RD29B* and *RD20* (Figure 5), as well as the fact that *CCoAOMT1* is highly expressed during seed germination (Figure S1), we further examined the seed germination in the presence of ABA to understand ABA sensitivity of *ccoaomt1* mutants. Before testing an ABA-mediated seed germination assay, we confirmed that all tested seeds were well-germinated and the seed defectiveness in *ccoaomt1* mutants were not observed compared to WT during seed germination in the absence of ABA (Figure 6, 0 μM ABA). Next, we analyzed seed germination on day 5 in the presence of ABA, which revealed that the number of opened cotyledons of *ccoaomt1* mutants were more than those of WT (Figure 6). The expression levels of *NCED3* and *ABA3* genes were not different between WT and *ccoaomt1* mutant plants (Figure 5E,F, 0 min). As ABA arrests seed germination [28], the WT seeds showed a delayed germination by increasing ABA levels (Figure 6B). Consistent to lower expression of both ABA responsive genes and biosynthetic genes, both *ccoaomt1-1* and *ccoaomt1-2* mutant seeds exhibit a less-delayed seed germination phenotype on higher concentration than 0.75 μM ABA compared with WT (Figure 6B). This result indicates that *CCoAOMT1* plays a role in plant response to ABA.

## 3. Discussion

It is known that CCoAOMT1 is a critical enzyme in lignin biosynthesis and its expression is regulated in response to various environmental stresses [4]. Therefore, we hypothesized that CCoAOMT1 is involved in environmental stress responses, such as drought. We confirmed that the expression of *CCoAOMT1* was strongly induced during salt, osmotic, and dehydration stresses (Figure 1). Connected to transcriptional regulation during dehydration treatment (Figure 1), we observed that *ccoaomt1-1* and *ccoamot1-2* mutants exhibited drought-sensitive phenotype with higher water loss (Figure 2). Proteome analysis of Yoshimura et al. (2008) showed that CCoAOMT was increased during both early and late stages of drought stress in wild watermelon (*Citrullus lanatus* sp.) [30]. Notably, we previously observed that the transcription of *CCoAOMT1* was significantly upregulated in salt-adapted cells [22]. Taken together, these results support that *CCoAOMT1* functions positively during drought and salt stresses. Drought stress inevitably results in oxidative damage due to the overproduction of ROS. ROS are the result of the partial reduction of atmospheric O_2_, such as superoxide radical (O_2_^−^) and hydrogen peroxide (H_2_O_2_), and possess oxidizing potential, with which unrestricted oxidation of the cellular components ultimately cause to cell death [31]. As inducing *CCoAOMT1* transcription by treatment of MV, *CCoAOMT1* null mutants exhibited less detoxification activity of MV-mediated ROS (Figure 3), as well as higher H_2_O_2_ accumulation in the leaves under drought stress conditions (Figure 4). These results suggest that *CCoAOMT1* is involved in ROS detoxification and the ROS signaling induced by drought stress.

Considered as a stress phytohormone, ABA in plants is dramatically induced by abiotic stresses such as salt and drought, contributing to being tolerant against these stresses [25]. Moreover, as drought-induced ABA influences ROS production and increases antioxidants, its positive ABA–ROS feedback loop results in higher ROS and ABA levels to control gene expression and mediates cellular responses to drought stress. More specifically, increased ABA levels stimulate the antioxidant system to protect cells from oxidative damage by over-accumulated ROS [32,33] and induce the expressions of ABA/drought-responsive genes [19,34].

Based on the observation about drought-sensitive phenotype of *ccoaomt1* mutant plants (Figure 2), we investigated that well-known dehydration responsive genes, including *RD20*, *RD29A*, *RD29B*, and *ERD1* were downregulated in *ccoaomt1* mutants (Figure 5A–D), and suggested that CCoAOMT1 is involved in response to drought stress. In addition, we figured out that the expression of ABA biosynthetic genes, *NCED3* and *ABA3*, were also downregulated under the drought conditions in *ccoaomt1* mutants (Figure 3E,F), suggesting that *ccoaomt1* mutants may have lower amounts of ABA during drought stress than WT.

Recently, RD20 was identified as AtCLO3, belonging to a small multigenic family of proteins, i.e., caleosins, that are thought to play roles in lipid degradation during seed germination [35]. RD20 plays a role in drought tolerance through control of stomatal opening under water deficit conditions rather than seed germination, as reported using a good ABA/drought-responsive marker [35,36]. As similar observations, such as induced gene expression of *CCoAOMT1* by ABA treatment (Figure 1E) and increased during seed germination (Figure S1), the *ccoaomt1* mutants showed reduced ABA sensitivity during germination compared to WT (Figure 6) [37]. Furthermore, *rd20* null mutants exhibit faster germination and more expanded green cotyledons during post-germination growth than WT in the presence of ABA, suggesting that RD20 is involved in enhancing sensitivity to endogenous ABA [36]. Paraquat treated *rd20* leaves exhibit higher degrees of cell death and 5–7-fold higher amounts of H_2_O_2_ compared to WT [36]. On the other hand, *RD20*-overexpressing plants exhibit counter phenotypes to respond on sensitivity to ABA and ROS compared to WT [35,36]. Overall, it is suggested that RD20 plays a protective role against drought stress by responding to ABA and regulating ROS accumulation, although the role of RD20 related to the relationship and/or interconnection pathways between ABA and ROS remains to be solved. In phenotypical views, *ccoaomt1* mutants exhibited similar physiological phenotype to that of *rd20* mutant, such as ABA sensitivity during seed germination and ROS accumulation during drought stress. These results indicate that CCoAOMT1 shows the same biological function as RD20 against drought stress and suggest that CCoAOMT1 functions as a positive regulator to respond to drought stress in ABA/ROS-mediated signaling pathways, although both proteins have different biochemical properties, molecular targets, and cellular functions.

We additionally observed the downregulated expression of dehydration responsive genes in ABA-independent pathway, such as *RD29A* and *ERD1* during dehydration in *ccoaomt1* mutants (Figure 5E,F) [19]. This indicates that the *CCoAOMT1* gene also follows the ABA-independent pathway in response to drought stress.

It is well-known that plant cells change cell wall composition to adapt to abiotic stresses, such as salt and drought stress [38]. The study using bermudagrass Tifton-85 showed that the lignin content was increased under drought conditions [39], and greater lignin content in the xylem improves resistance to drought stress due to cell wall deposition [38,40]. Although the molecular mechanism for changes in cell wall dynamics by salt or drought stresses is unclear, the chemical reaction requires ROS to crosslink with phenolics [38]. The structure of xylem cell walls is modified in response to different environmental stimuli and influences xylem transport patterns, which controls water flow dynamics and contributes to systemic and fine-tuned regulation of plant growth under environmental stress conditions [41,42]. The dominant expression of *CCoAOMT1* in xylem tissues based on the analysis using an *Arabidopsis* plant expressing the GUS report gene driven by *CCoAOMT1* promoter and the reduced lignin content in *ccoaomt1* mutant [21] suggested that CCoAOMT1 may influence secondary cell wall formation of xylem. We speculate that the loss of CCoAOMT1 function affects lignin biosynthesis in the secondary cell wall of the xylem and may further result in alterations in the physical properties of the xylem transport system.

Conclusively, our results, including the gene expression patterns of *CCoAOMT1*, as well as phenotypic characteristics of *ccoaomt1* mutants, suggest that *CCoAOMT1* requires to be tolerant against drought stress by regulating cell wall lignification as well as by modulating both ABA and ROS signaling pathways.

## 4. Materials and Methods

### 4.1. Plant Growth and Stress Conditions

In our experiments, *Arabidopsis thaliana* (ecotype Col-0) was used for wild-type and T-DNA inserted *ccoaomt1* mutants, *ccoaomt1-1* and *ccoaomt1-2,* which were used as descried by Chun et al. [22]. Plants were grown in a 1/2 Murashige and Skoog (MS) medium (1.0% (*w*/*v*) sucrose, 0.6% (*w*/*v*) agar, pH 5.7) in a growth chamber (23 °C, 60% RH, 150 µE/M2/s fluorescent illumination) in a 16 h light/8 h dark cycle. For the qRT-PCR analysis, 10-day-old wild-type (Col-0) seedlings were treated with various stresses (100 mM NaCl, 10 mM LiCl, 100 mM mannitol, drought, 100 µM ABA, and 10 µM MV) at indicated time points. For the drought stress treatments, we used a growth chamber. At least 36 plants withheld water from two-week-old grown plants in soil pots for 11 days, and the survival ratio (%) was calculated by counting differential morphological plants one day after rewatering (% = greened plants(n)/total plants (n) × 100). For MV treatment, at least 10-day-old seedlings were transferred into 1/2 MS medium in the presence of indicated concentration of MV (Sigma-Aldrich, Saint Louis, MO, USA). Photographs were taken 2 days after transfer.

For the germination assay, 36 seeds were sown on a 1/2 MS agar medium supplemented with different concentrations of ABA, and the numbers of opened green cotyledons were counted on the 5th day, wherein we calculated the germination rate (%).

### 4.2. Quantitative Real-Time PCR (qRT-PCR) Analyses

Total RNAs were extracted from 10-day-old seedlings using the RNeasy Kit (Qiagen) following the manufacturer’s instructions. After treating DNase I for eliminating contaminated gDNA, the first-strand cDNA was synthesized from 1 µg of total RNA using a cDNA synthesis kit (Thermo Fisher Scientific, Waltham, MA, USA). The QuantiSpeed SYBR No-Rox Mix (PhileKorea, Seoul, Korea) was used for the qRT-PCR reactions as follows: 50 °C for 10 min, 95 °C for 10 min, and 50 cycles of 95 °C for 15 s, 60 °C for 15 s, and 72 °C for 15 s. The relative expression of genes was normalized with the expression of *TUBULIN2* and calculated through the CFX Manager software (Bio-Rad Laboratories, Hercules, CA, USA). The specific primers of genes used for the qRT-PCR analysis are listed in Supplementary Table S1.

### 4.3. Water Loss Assay

The leaves of 3-week-old plants grown in soil were detached and weighed immediately. The detached leaves were placed on a plate at room temperature and weighed at various time points. The loss of fresh weight was calculated as a percentage of the initial weight of the plant.

### 4.4. Chlorophyll Measurement

The chlorophyll contents in leaves were measured according to the protocol of Faragó et al. [43]. The 10–20 mg of tissues were pulverized in 1 mL of 80% acetone. The absorbance of supernatant solution was measured at A_664_ nm and A_647_ nm. The total chlorophyll content in leaves was calculated using the following formula: Amount of chlorophyll (µg mg^−1^) = (7.17 × A_664_) + (17.67 × A_647_)/dilution factor/(mg, fresh weight of leaves). The values of total chlorophyll indicated means ± SD of five seedlings from three independent experiments.

### 4.5. H_2_O_2_ Staining and Its Content Measurement

The 3,3′-diaminobenzidine (DAB; Sigma-Aldrich, Saint Louis, MO, USA) staining was detected to accumulate hydrogen peroxide (H_2_O_2_) in plant tissues as a dark brown precipitate [44]. The DAB solution (1 mg/mL) was dissolved in 10 mM Na_2_HPO_4_ and reduced pH 3.0 with 0.2 M HCl. The 10-day-old seedlings were immersed in DAB solution and shaken for 8 h or more in the dark. Chlorophyll was removed with a bleaching solution (ethanol:acetic acid:glycerol = 3:1:1) and then boiled for 15 min.

The H_2_O_2_ was measured from at least five seedlings using the Amplex Red Hydrogen Peroxide/Peroxidase Assay Kit (Thermo Fisher Scientific, Waltham, MA, USA) following the manufacturer’s instructions. Fluorescence was determined by excitation at 530 nm and emission at 590 nm using the SpectraMax GEMINI XPS spectrofluorometer (Molecular Devices, San Jose, CA, USA). H_2_O_2_ contents were analyzed as described previously [45].

### 4.6. Statistical Analyses

Statistical analyses, including Student’s *t*-test, were performed using Excel 2010. The qRT-PCR analysis was performed in three independent experiments and the average values of 2^ΔΔCT^ were used to determine the differences. Data are indicated as means ± SD. Error bars indicate standard deviation (SD).

## Supplementary Materials

The following are available online at https://www.mdpi.com/article/10.3390/plants10050831/s1, Table S1: Primers used for qRT-PCR, Figure S1: Expression patterns of *CCoAOMT1* during seed germination.

## References

[1] Gull A., Ahmad Lone A., Ul Islam Wani N. (2019). Biotic and abiotic stresses in plants. Abiotic Biot. Stress Plants.

[2] Bechtold U., Field B. (2018). Molecular mechanisms controlling plant growth during abiotic stress. J. Exp. Bot..

[3] Yadav S., Modi P., Dave A., Vijapura A., Patel D., Patel M. (2020). Effect of abiotic stress on crops. Sustain. Crop. Prod..

[4] Le Gall H., Philippe F., Domon J.M., Gillet F., Pelloux J., Rayon C. (2015). Cell wall metabolism in response to abiotic stress. Plants (Basel).

[5] Hamann T. (2015). The plant cell wall integrity maintenance mechanism-Concepts for organization and mode of action. Plant Cell Physiol..

[6] Moura J.C.M.S., Bonine C.A.V., de Oliveira Fernandes Viana J., Dornelas M.C., Mazzafera P. (2010). Abiotic and biotic stresses and changes in the lignin content and composition in plants. J. Integr. Plant Biol..

[7] Lee B.R., Kim K.Y., Jung W.J., Avice J.C., Ourry A., Kim T.H. (2007). Peroxidases and lignification in relation to the intensity of water-deficit stress in white clover (*Trifolium repens* L.). J. Exp. Bot..

[8] Yang L., Wang C.C., Guo W.D., Li X.B., Lu M., Yu C.L. (2006). Differential expression of cell wall related genes in the elongation zone of rice roots under water deficit. Russ. J. Plant Physiol..

[9] Miller G., Suzuki N., Ciftci-Yilmaz S., Mittler R. (2010). Reactive oxygen species homeostasis and signalling during drought and salinity stresses. Plant Cell Environ..

[10] Cruz De Carvalho M.H. (2008). Drought stress and reactive oxygen species: Production, scavenging and signaling. Plant Signal. Behav..

[11] Cerny M., Habanova H., Berka M., Luklova M., Brzobohaty B. (2018). Hydrogen peroxide: Its role in plant biology and crosstalk with signalling networks. Int. J. Mol. Sci..

[12] You J., Chan Z. (2015). Ros regulation during abiotic stress responses in crop plants. Front. Plant Sci..

[13] Nambara E., Marion-Poll A. (2005). Abscisic acid biosynthesis and catabolism. Annu. Rev. Plant Biol..

[14] Vishwakarma K., Upadhyay N., Kumar N., Yadav G., Singh J., Mishra R.K., Kumar V., Verma R., Upadhyay R.G., Pandey M. (2017). Abscisic acid signaling and abiotic stress tolerance in plants: A review on current knowledge and future prospects. Front. Plant Sci..

[15] Sreenivasulu N., Harshavardhan V.T., Govind G., Seiler C., Kohli A. (2012). Contrapuntal role of ABA: Does it mediate stress tolerance or plant growth retardation under long-term drought stress?. Gene.

[16] Hossain M.A., Wani S.H., Bhattacharjee S., Burritt D.J., Tran L.S.P. (2016). Drought stress tolerance in plants, vol 2: Molecular and genetic perspectives. Drought Stress Toler. Plants Vol 2 Mol. Genet. Perspect..

[17] Chaves M.M., Maroco J.P., Pereira J.S. (2003). Understanding plant responses to drought—From genes to the whole plant. Funct. Plant Biol..

[18] Roychoudhury A., Paul S., Basu S. (2013). Cross-talk between abscisic acid-dependent and abscisic acid-independent pathways during abiotic stress. Plant Cell Rep..

[19] Liu C., Zhang X., Zhang K., An H., Hu K., Wen J., Shen J., Ma C., Yi B., Tu J. (2015). Comparative analysis of the Brassica napus root and leaf transcript profiling in response to drought stress. Int. J. Mol. Sci..

[20] Fellenberg C., Van Ohlen M. (2012). The role of CCoAOMT1 and COMT1 in Arabidopsis anthers. Planta.

[21] Do C.T., Pollet B., Thévenin J., Sibout R., Denoue D., Barrière Y., Lapierre C., Jouanin L. (2007). Both caffeoyl Coenzyme A 3-O-methyltransferase 1 and caffeic acid O-methyltransferase 1 are involved in redundant functions for lignin, flavonoids and sinapoyl malate biosynthesis in Arabidopsis. Planta.

[22] Chun H.J., Baek D., Cho H.M., Lee S.H., Jin B.J., Yun D.J., Hong Y.S., Kim M.C. (2019). Lignin biosynthesis genes play critical roles in the adaptation of Arabidopsis plants to high-salt stress. Plant Signal. Behav..

[23] Barbagallo R.P., Oxborough K., Pallett K.E., Baker N.R. (2003). Rapid, noninvasive screening for perturbations of metabolism and plant growth using chlorophyll fluorescence imaging. Plant Physiol..

[24] Hariadi Y., Marandon K., Tian Y., Jacobsen S.E., Shabala S. (2011). Ionic and osmotic relations in quinoa (Chenopodium quinoa Willd.) plants grown at various salinity levels. J. Exp. Bot..

[25] Chen K., Li G.J., Bressan R.A., Song C.P., Zhu J.K., Zhao Y. (2020). Abscisic acid dynamics, signaling, and functions in plants. J. Integr. Plant Biol..

[26] Iuchi S., Kobayashi M., Taji T., Naramoto M., Seki M., Kato T. (2001). Regulation of drought tolerance by gene manipulation of 9- cis -epoxycarotenoid dioxygenase, a key enzyme in abscisic acid biosynthesis in Arabidopsis. Plant J..

[27] Li Y., Zhang J., Zhang J., Hao L., Hua J., Duan L., Zhang M., Li Z. (2013). Expression of an Arabidopsis molybdenum cofactor sulphurase gene in soybean enhances drought tolerance and increases yield under field conditions. Plant Biotechnol. J..

[28] Daszkowska-Golec A. (2011). Arabidopsis seed germination under abiotic stress as a concert of action of phytohormones. Omics. J. Integr. Biol..

[29] Finkelstein R.R., Gampala S.S.L., Rock C.D. (2002). Abscisic acid signaling in seeds and seedlings. Plant Cell.

[30] Yoshimura K., Masuda A., Kuwano M., Yokota A., Akashi K. (2008). Programmed proteome response for drought avoidance/tolerance in the root of a C3 xerophyte (wild watermelon) under water deficits. Plant Cell Physiol..

[31] Chaki M., Begara-Morales J.C., Barroso J.B. (2020). Oxidative stress in plants. Antioxidants.

[32] Hu X., Zhang A., Zhang J., Jiang M. (2006). Abscisic acid is a key inducer of hydrogen peroxide production in leaves of maize plants exposed to water stress. Plant Cell Physiol..

[33] Arve L.E., Carvalho D.R.A., Olsen J.E., Torre S. (2014). ABA induces H2O2 production in guard cells, but does not close the stomata on vicia faba leaves developed at high air humidity. Plant Signal. Behav..

[34] Singh D., Laxmi A. (2015). Transcriptional regulation of drought response: A tortuous network of transcriptional factors. Front. Plant Sci..

[35] Aubert Y., Vile D., Pervent M., Aldon D., Ranty B., Simonneau T., Vavasseur A., Galaud J.P. (2010). RD20, a stress-inducible caleosin, participates in stomatal control, transpiration and drought tolerance in Arabidopsis thaliana. Plant Cell Physiol..

[36] Blée E., Boachon B., Burcklen M., Le Gaé M., Abdulsamie H., Heintz D., Ehlting J., Herrfurth C., Feussner I., Bessoule J.J. (2014). The reductase activity of the arabidopsis caleosin RESPONSIVE TO DESSICATION20 mediates gibberellin-dependent flowering time, abscisic acid sensitivity, and tolerance to oxidative stress1[w]. Plant Physiol..

[37] del Rodríguez-Gacio M.C., Matilla-Vázquez M.A., Matilla A.J. (2009). Seed dormancy and ABA signaling: The breakthrough goes on. Plant Signal. Behav..

[38] Tenhaken R. (2015). Cell wall remodeling under abiotic stress. Front. Plant Sci..

[39] Saha U. (2016). The effect of drought on lignin content and digestibility of Tifton-85 and coastal bermudagrass (*Cynodon dactylon* L.) Hays Produced in Georgia. Int. J. Appl. Agric. Sci..

[40] Pereira L., Domingues-Junior A.P., Jansen S., Choat B., Mazzafera P. (2018). Is embolism resistance in plant xylem associated with quantity and characteristics of lignin?. Trees Struct. Funct..

[41] Taylor-Teeples M., Lin L., De Lucas M., Turco G., Toal T.W., Gaudinier A., Young N.F., Trabucco G.M., Veling M.T., Lamothe R. (2015). An arabidopsis gene regulatory network for secondary cell wall synthesis. Nature.

[42] Endo S., Iwai Y., Fukuda H. (2019). Cargo-dependent and cell wall-associated xylem transport in Arabidopsis. New Phytol..

[43] Faragó D., Sass L., Valkai I., Andrási N., Szabados L. (2018). Plantsize offers an affordable, non-destructive method to measure plant size and color in vitro. Front. Plant Sci..

[44] Daudi A., O’Brien J.A. (2012). Detection of hydrogen peroxide by DAB staining in arabidopsis leaves. Bio-Protocol.

[45] Grellet Bournonville C.F., Díaz-Ricci J.C. (2011). Quantitative determination of superoxide in plant leaves using a modified NBT staining method. Phytochem. Anal..

