# Supplementary material for: Arabidopsis CCoAOMT1 Plays a Role in Drought Stress Response via ROS- and ABA-Dependent Manners
Source: Plants (Basel). 2021 Apr 21;10(5):831. doi: 10.3390/plants10050831 (PMC8143326; doi:10.3390/plants10050831)
Supplement: Supplementary file 1 [file plants-10-00831-s001.zip › plants-1195276-supplementary.pdf]

Supporting information

Arabidopsis CCoAOMT1 plays a role in drought stress response via ROS- and ABA-dependent manners

Hyun Jin Chun [1,¶], Lack Hyeon Lim [2,¶], Mi Sun Cheong [1,¶], Dongwon Baek [3,¶], Mi Suk Park [3], Hyun Min Cho [2], Su Hyeon Lee [2], Byung Jun Jin [2], Dong Hyeon No [2], Ye Jin Cha [2], Yong Bok Lee [1,2], Jong Chan Hong [3], Dae-Jin Yun [4], and Min Chul Kim [1,2,3,*]

[1] Institute of Agriculture & Life Science, Gyeongsang National University, Jinju, Korea; hj_chun@hanmail.net (H.J.C), mscheong@gnu.ac.kr (M.S.C.), yblee@gnu.ac.kr (Y.B.L.)

[2] Division of Applied Life Science (BK21 Four), Gyeongsang National University, Jinju, Korea; dlafkrgus@gnu.ac.kr (L.H.L), hmcho86@gnu.ac.kr (H.M.C), leesuhyeon86@gmail.com (S.H.L), scv5789@naver.com (B.J.J), no0513w@naver.com (D.H.N), cdw3280@naver.com (Y.J.C)

[3] Plant Molecular Biology and Biotechnology Research Center, Gyeongsang National University, Jinju, Korea; dw100@hanmail.net (D.B), misugip@hanmail.net (M.S.P), jchong@gnu.ac.kr (J.C.H)

[4] Department of Biomedical Science & Engineering, Konkuk University, Seoul, Korea; djyun@konkuk.ac.kr (D-J.Y)

[¶] These authors contributed equally in this study

* Correspondence: mckim@gnu.ac.kr, Tel.: +82-55-772-1874 (M.C.K.)

Supplementary Materials:

Table S1: Primers used for qRT-PCR

Figure S1: Expression patterns of *CCoAOMT1* during seed germination.

Supplementary Table S1. Primers used for qRT-PCR

Gene	Primer name	Sequence (5'→3')
CCoAOMT1 (AT4G34050)	CCoAOMT1-qRT-F	5'-CTGGCTATGGATGTCAACAGAG-3'
	CCoAOMT1-qRT-R	5'-ATCAAGAACGGGAAGAGCAG-3'
RD29B (AT5G52300)	RD29B-qRT-F	5'-GTGAAGATGACTATCTCGGTGG-3'
	RD29B-qRT-R	5'-CACCACTGAGATAATCCGATCC-3'
RD20 (AT2G33380)	RD20-qRT-F	5'-TTAGCTCCGGTCACCAGTCA-3'
	RD20-qRT-R	5'-CATGTATGGTTTTGGTAATGTTTCC-
RD29A (AT5G52310)	RD29A-qRT-F	5'-CCTGAAGTGATCGATGCACCAG-3'
	RD29A-qRT-R	5'-TGGTGTAATCGGAAGACACGAC-3'
ERD1 (AT5G51070)	ERD1-qRT-F	5'-CAGATGATGGAGATCTTGAA-3'
	ERD1-qRT-R	5'-TGCGATCGATGTTTTGT-3'
ABA3 (AT1G16540)	ABA3-qRT-F	5'-CTTCAGGCTTTACGACATGG-3'
	ABA3-qRT-R	5'-CCAAACCAAGAGCCATCA-3'
NCED3 (AT3G14440)	NCED3-qRT-F	5'-TCCAGCTCTTCATTTCCCTAA-3'
	NCED3-qRT-R	5'-CGGCCATTGAAATAGACCAA-3'
TUBULIN2 (AT5G62690)	TUBULIN2-qRT-F	5'-TGGCATCAACTTTCATTGGA-3'
	TUBULIN2-qRT-R	5'-ATGTTGCTCTCCGCTTCTGT-3'

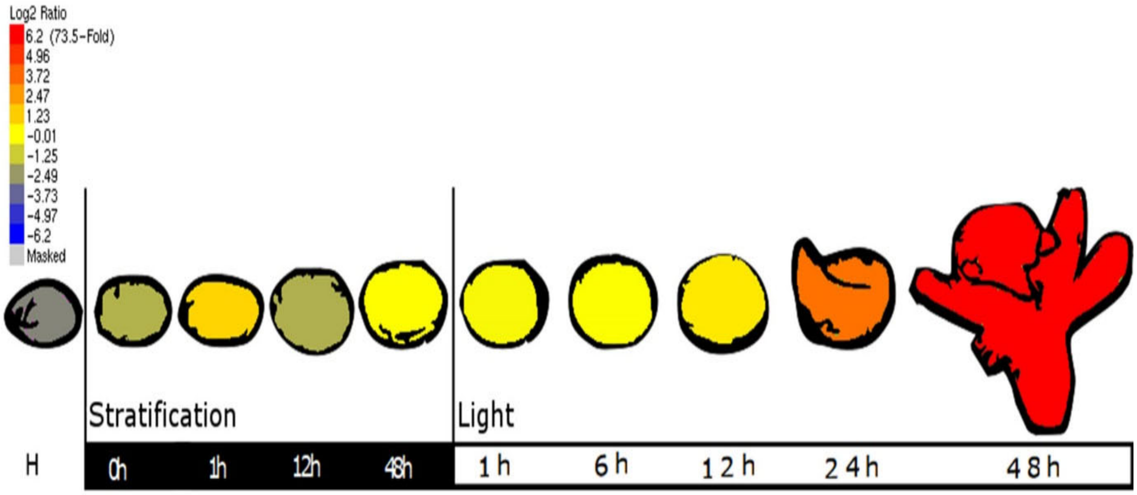

Figure S1. Expression patterns of *CCoAOMT1* during seed germination.

Relative expression patterns of *CCoAOMT1* in data source of germination using *Arabidopsis* eFP Browser at BAR website (http://bar.utoronto.ca/efp/cgi-bin/efpWeb.cgi).
